# A phase 3, randomised, placebo-controlled study of erenumab for the prevention of chronic migraine in patients from Asia: the DRAGON study

**DOI:** 10.1186/s10194-022-01514-9

**Published:** 2022-11-21

**Authors:** Shengyuan Yu, Byung-Kun Kim, Hebo Wang, Jiying Zhou, Qi Wan, Tingmin Yu, Yajun Lian, Michal Arkuszewski, Laurent Ecochard, Shihua Wen, Fangfang Yin, Zheng Li, Wendy Su, Shuu-Jiun Wang

**Affiliations:** 1 Chinese PLA General Hospital, Beijing, China; 2 Nowon Eulji Medical Center, Eulji University School of Medicine, Seoul, Korea; 3 Hebei General Hospital, Shijiazhuang, China; 4 The First Affiliated Hospital of Chongqing Medical University, Chongqing, China; 5 Jiangsu Province Hospital, Nanjing, China; 6 The Second Hospital of Jilin University, Changchun, China; 7 The First Affiliated Hospital of Zhengzhou University, Zhengzhou, China; 8 Novartis Pharma AG, Basel, Switzerland; 9 Novartis Pharmaceuticals Corporation, East Hanover, NJ USA; 10 China Novartis Institutes for Biomedical Research Co., Ltd, Shanghai, China; 11 Neurological Institute, Taipei Veterans General Hospital, Taipei, Taiwan; 12 Brain Research Center and College of Medicine, National Yang Ming Chiao Tung University, Taipei, Taiwan

**Keywords:** Asian, Calcitonin gene-related peptide, Chronic migraine, Efficacy, Erenumab, Monoclonal antibodies, Clinical trial, Safety

## Abstract

**Background:**

DRAGON was a phase 3, randomised, double-blind, placebo-controlled study which evaluated the efficacy and safety of erenumab in patients with chronic migraine (CM) from Asia not adequately represented in the global pivotal CM study.

**Methods:**

DRAGON study was conducted across 9 Asian countries or regions including mainland China, India, the Republic of Korea, Malaysia, the Philippines, Singapore, Taiwan, Thailand, and Vietnam. Patients (*N* = 557) with CM (aged 18–65 years) were randomised (1:1) to receive once-monthly subcutaneous erenumab 70 mg or matching placebo for 12 weeks. The primary endpoint was the change in monthly migraine days (MMD) from baseline to the last 4 weeks of the 12-week double-blind treatment phase (DBTP). Secondary endpoints included achievement of ≥ 50% reduction in MMD, change in monthly acute headache medication days, modified migraine disability assessment (mMIDAS), and safety. Study was powered for the primary endpoint of change from baseline in MMD.

**Results:**

At baseline, the mean (SD) age was 41.7 (± 10.9) years, and 81.5% (n = 454) patients were women. The mean migraine duration was 18.0 (± 11.6) years, and the mean MMD was 19.2 (± 5.4). 97.8% (n = 545) randomised patients completed the DBTP. Overall, demographics and baseline characteristics were balanced between the erenumab and placebo groups except for a slightly higher proportion of women in the placebo group. At Week 12, the adjusted mean change from baseline in MMD was − 8.2 days for erenumab and − 6.6 days for placebo, with a statistically significant difference for erenumab versus placebo (adjusted mean difference vs placebo: − 1.57 [95%CI: − 2.83, − 0.30]; *P* = 0.015). A greater proportion of patients treated with erenumab achieved ≥ 50% reduction in MMD versus placebo (47.0% vs 36.7%, *P* = 0.014). At Week 12, greater reductions in monthly acute headache medication days (− 5.34 vs − 4.66) and mMIDAS scores (− 14.67 vs − 12.93) were observed in patients treated with erenumab versus placebo. Safety and tolerability profile of erenumab was comparable to placebo, except the incidence of constipation (8.6% for erenumab vs 3.2% for placebo).

**Conclusion:**

DRAGON study demonstrated the efficacy and safety of erenumab 70 mg in patients with CM from Asia. No new safety signals were observed during the DBTP compared with the previous trials.

**Trial registration:**

NCT03867201

**Supplementary Information:**

The online version contains supplementary material available at 10.1186/s10194-022-01514-9.

## Introduction

Migraine is the second leading cause of years lived with disability [1, 2]. In 2019, an estimated 1.1 billion cases of migraine were reported globally, with the highest prevalence in China (188.9 million) followed by Taiwan and the Republic of Korea (3.4 million each) amongst East Asian countries [3]. Migraine interferes with the everyday activities of individuals and their family members [4] and is associated with a high socio-economic burden [5, 6].

Migraine is classified based on the headache frequency as episodic migraine (EM: < 15 headache days/month, which on some days is migraine) [7] and chronic migraine (CM: ≥ 15 headache days/month for 3 months, including ≥ 8 migraine days/month) [8]. CM is a highly burdensome disorder because the burden of migraine increases with an increase in the headache frequency [9].

Currently, limited oral preventive treatment options are available for patients with migraine in Asia. The available oral preventive therapies are not fully efficacious or are poorly tolerated [10], which has led to low adherence rates [11, 12]. Hence, innovative and well-tolerated treatments are necessary for patients suffering from migraine. In East Asia, a low level of disease awareness and use of prescription medication are barriers to the effective management of migraine [13]. Migraine has been commonly managed with the use of complementary medicines in Asia, whereas evidence supporting their use is minimal and often inadequate [14].

At present, treatment guidelines for migraine prevention are available in mainland China [15], Taiwan [16], and Republic of Korea [17]. In China, most of the available treatments are not approved for migraine prevention and off-label use of medications is common in clinical practice [18], resulting in a low proportion of patients receiving preventives specifically developed for migraine treatment. For instance, in North America and Europe, topiramate is widely used for the preventive treatment of migraine [19], but topiramate is not approved in China for migraine prevention [20]. Onabotulinum toxin type A is also available but not approved for the migraine prevention in China [20]. Currently, flunarizine is the only approved drug for migraine prevention in China [15]. According to a retrospective analysis of the China Health Insurance Research Association (CHIRA) database, patients with migraine who were prescribed at least one preventive medication were as low as 15.0%, with a majority (88.3%) receiving calcium channel blockers (primarily flunarizine) [21]. Access to effective treatments, therefore, remains a significant unmet medical need for migraine prevention in Asia.

The monoclonal antibodies targeting the calcitonin gene-related peptide (CGRP) pathway (ligand or its receptor) represent a new class of targeted mechanism-specific preventive treatment for migraine [22, 23]. Erenumab is the first and only fully human monoclonal antibody that targets and selectively blocks the canonical CGRP receptor [24]. Clinical trials conducted in the US, the European Union (EU), and Japan have demonstrated the efficacy and safety of erenumab (70 mg and 140 mg) in the prevention of migraine in adult patients with EM and CM [25–29]. Furthermore, the recent EMPOwER phase 3 study [30] demonstrated the efficacy and safety of erenumab in patients with EM primarily from Asia.

Although erenumab is now approved for the preventive treatment of migraine in adult patients in many countries [31–34], there is a need to obtain data on erenumab from China and other Asian countries not adequately represented in the global pivotal CM study [28]. The DRAGON study was performed to evaluate the efficacy and safety of once-monthly subcutaneous erenumab 70 mg compared with placebo in patients with CM from China and other Asian countries.

## Methods

### Study design

**Fig. 1:**
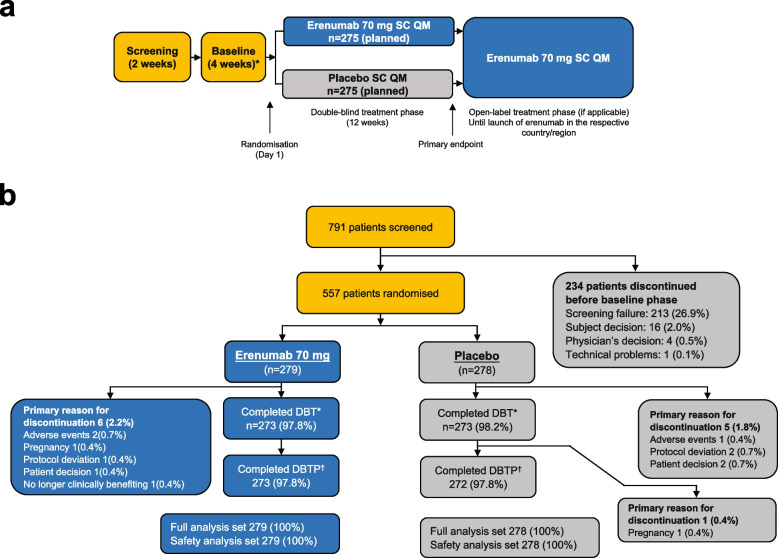
**a** Study design. Note: For patients not entering the OLTP, the safety follow-up visit occurred 12 weeks after the last dose of the DBTP. In the selected countries or regions, patients completing the DBTP on the study drug were eligible to participate in the OLTP (until launch of erenumab in the respective country or region). The study was powered only for the primary endpoint assessed in the overall population with an alpha level of 0.05. The endpoints were assessed as change from baseline over the last 4 weeks of the DBTP. * Duration of baseline was up to 35 days (5 weeks) due to COVID-19, with the last 28 days of this period considered for the assessment of headache frequency (MMD). DBTP, double-blind treatment phase; OLTP, open-label treatment phase; QM, once monthly; SC, subcutaneous. **b** Patient disposition in the DBTP. Note: *A patient is defined as a completer for the DBT if the individual had received the Week 8 dose (erenumab or placebo). ^†^ A patient is defined as completer for the DBTP, if the individual had completed all scheduled visits in the DBTP, including Week 12 visit, or entered in the OLTP. For patients who continued to OLTP, the cut-off was end of treatment. For patients who entered into the safety follow-up after double-blind treatment, the cut-off is 11-Aug-2021 or end of study whichever is the earliest. Screened were those patients who had signed an informed consent form. Full analysis set comprised all randomised patients. DBT, double-blind treatment; DBTP, double-blind treatment phase; OLTP, open-label treatment phase

DRAGON (ClinicalTrials.gov Identifier: NCT03867201) was a phase 3, 12-week, randomised, double-blind, placebo-controlled study of erenumab (70 mg; Fig. 1a). The study was conducted between August 2019 and August 2021 at 64 sites in 9 Asian countries or regions including mainland China, India, the Republic of Korea, Malaysia, the Philippines, Singapore, Taiwan, Thailand, and Vietnam. The study comprised the following periods: a screening period (up to 2 weeks) to assess initial eligibility; a baseline period (up to 5 weeks) to assess baseline diary compliance and headache frequency for the final eligibility before randomisation and dosing; a 12-week, double-blind treatment phase (DBTP); and an open-label treatment phase (OLTP) until the launch of erenumab in the respective country or region (Fig. 1a). Patients discontinuing treatment during the DBTP and willing to return for the subsequent scheduled safety follow-up visits were followed for 12 weeks after the last visit, and patients completing the DBTP but not entering into the OLTP were followed for 8 weeks after treatment completion. Here we report the efficacy and safety results obtained from the DBTP.

Patients with CM were randomised (1:1) to receive once-monthly subcutaneous injections of either erenumab 70 mg or placebo for 12 weeks. Patients were randomised on Day 1 using interactive response technology (IRT). A patient randomisation list was produced by the IRT provider using a validated system that automates the random assignment of patient numbers to randomisation numbers. The members of the randomisation group reviewed and approved the randomisation scheme for patients. Randomisation was stratified by prior preventive migraine treatment failure (yes vs no) and medication overuse status (yes vs no) according to the planned randomisation ratio within each stratum.

Prior preventive migraine treatment failure was defined as either efficacy or tolerability failure in one of the pre-defined medication categories (Supplementary Table 1). Prior preventive migraine treatment failure status was based on historical data. Patients with co-existing medication overuse of triptans, ergot derivatives, analgesics, and combination drug use were allowed in the study. Medication overuse was considered present if any of the following criteria were met during the baseline period: ≥ 15 days of simple analgesics; ≥ 10 days of triptans; ≥ 10 days of ergot derivatives; ≥ 10 days of combination therapy intake of any combination of ergot derivatives, triptans, combination-analgesic medications or simple analgesics; and ≥ 10 days of total consecutive therapy intake of multiple medications including simple analgesics, triptans or ergot derivatives, each of which may not be overused individually. The medication overuse status was based on the medication reported during the baseline period.

### Ethical considerations

The study protocol, protocol amendments, and informed consent forms were reviewed and approved by the Institutional Review Board/Independent Ethics Committee and health authorities at each study centre. This study was conducted in accordance with the International Council for Harmonisation E6 guideline for good clinical practice (GCP), applicable local regulations and guidelines, and the ethical principles laid down in the Declaration of Helsinki.

All patients provided written informed consent before undergoing any study-specific procedures.

### Study participants

Eligible patients were adults aged 18–65 years with a history of CM with or without aura for at least 12 months before screening as defined by the International Classification of Headache Disorders, 3^rd^ edition (ICHD-3) [8]. Of note, having aura or not was based on medical records and/or self-report (Supplementary Table 2). Eligibility for randomisation was based on migraine frequency and eDiary compliance. Patients with a history of ≥ 15 headache days/month, of which ≥ 8 headache days met criteria as migraine days during the baseline period (all based on the eDiary calculations), and who had demonstrated at least 80% compliance with the eDiary during the baseline period were included. Patients were allowed to continue on “best supportive care” as rescue medication, which included both pharmacologic interventions (i.e., abortive treatments for acute attacks) and non-pharmacologic interventions (e.g. biofeedback, psychotherapy, acupuncture or other locally accepted and endorsed interventions for migraine). The chronically administered ‘best supportive care’ was recommended for all patients and had to be on a stable regimen for at least 1 month before start of the baseline period and throughout the study. Concomitant therapies with possible migraine prophylactic effects taken for indications other than migraine must have been administered at a stable dose within the 3 months prior to the start of the baseline period and throughout the study.

Patients were not eligible to participate in the study if they were older than 50 years at migraine onset. Other key exclusion criteria were as follows: a history of cluster or hemiplegic migraine headache; CM with continuous pain; unable to differentiate migraine from other headaches; opioid and/or opioid-containing analgesic (for > 4 days per month) or butalbital-containing analgesic (for > 2 days per month) for any indication within one month before the start of or during the baseline period; prior migraine preventive treatment failure in > 3 medication categories (categories provided in Supplementary Table 2); prior botulinum toxin A treatment in the head/neck region within 4 months before the start of or during the baseline period, active chronic pain syndromes (such as fibromyalgia and chronic pelvic pain), or other medical conditions (Supplementary Table 2). Pregnant or nursing (lactating) women, and women of childbearing potential were also excluded. The full eligibility criteria are presented in Supplementary Table 2.

### Endpoints and assessments

During baseline and DBTP, patients recorded headaches and headache medication information daily using the eDiary device. The eDiary device was assigned to patients after completion of the initial screening, and the data were collected up to the end of Core Period (Week 12 visit) or early discontinuation. Patients received training from the study site staff on how to use the eDiary for daily reporting and the study site staff reviewed eDiary compliance with the patient at each visit. The monthly migraine days (MMD) were calculated based on data recorded in eDiary.

The primary endpoint was the change from baseline in MMD during the last 4 weeks of the 12-week treatment period (i.e., Weeks 9–12). In this study, a migraine day was defined as any calendar day on which the patient experienced a qualified migraine headache (onset, continuation, or recurrence of the migraine headache). A qualified migraine headache was defined as a migraine with or without aura, lasting for ≥ 4 continuous hours, and meeting at least one of the following criteria: (a) ≥ 2 of the following pain features: unilateral, throbbing, moderate-to-severe, exacerbated with exercise/physical activity or (b) ≥ 1 of the following associated symptoms: nausea and/or vomiting, photophobia and phonophobia. If the patient took any acute medication (simple analgesics [non-steroidal anti-inflammatory drugs, acetaminophen], combination analgesics, triptans, or ergot derivatives) during aura, or to treat a moderate or severe headache on a calendar day, then it was counted as a migraine day regardless of the duration and pain features/associated symptoms.

Secondary endpoints were the achievement of ≥ 50% reduction in MMD from baseline, change in monthly acute headache medication days from baseline, and change in the modified migraine disability assessment (mMIDAS; see Supplementary Table 3) scores from baseline as assessed over the last 4 weeks of the DBTP (i.e., Weeks 9–12). Safety was assessed by the incidence of adverse events (AEs), serious AEs (SAEs), clinically important changes in clinical laboratory values and vital signs. Safety assessment also included the number and percentage of patients who developed anti-erenumab antibodies (binding and/or neutralizing) during the DBTP. Only the blood samples positive for binding antibodies were tested for neutralizing antibodies.

The Medical Dictionary for Regulatory Activities (MedDRA) Version 24.0 was used to code AEs to a system organ class and a preferred term. AEs were graded using the Common Terminology Criteria for Adverse Events (CTCAE) Version 4.03.

### Statistical analysis

The full analysis set included all patients who were randomised in the study. Patients were analysed according to their randomised treatment. Efficacy analyses were performed on the full analysis set. The safety analysis set comprised all randomised patients who received at least one dose of the investigational product. Patients were analysed based on the actual treatment received. Safety analyses were performed based on the safety analysis set.

Here, we report data from the 12-week DBTP, including safety follow-up for patients who did not enter the OLTP or discontinued during the DBTP. The patient demographic and baseline disease characteristics were summarised using descriptive statistics for the full analysis set. The mean, median, standard deviation (SD ±), minimum, and maximum are presented for continuous variables, and the number and percentage of patients in each category were presented as categorical variables for each treatment group and all patients (total).

The primary efficacy endpoint (change from baseline in MMD) was analysed using a generalised linear mixed effects repeated measures model based on observed monthly data during the treatment period. The model included treatment groups, scheduled visit, interaction of treatment group with scheduled visit, and the stratification factors (random) with baseline values as covariates. An unstructured covariance structure was assumed. The least-squares mean (LSM) for each treatment group, standard error (SE) and its associated 95% confidence interval (CI), the difference of LSMs compared with the placebo group and the associated 95% CI for the difference, as well as the nominal two-sided *P*-values, were tabulated by visit and treatment. The study was only powered for the primary endpoint of change from baseline in MMD during the last 4 weeks of the DBTP (Week 12).

The secondary efficacy endpoints (change from baseline in monthly acute headache medication days and mMIDAS) were analysed using the generalised linear mixed effects repeated measures model similar to the primary endpoint and reported in the same manner. The dichotomous secondary efficacy endpoint (proportion of patients achieving ≥ 50% reduction in MMD) was analysed by the Cochran–Mantel–Haenszel test, with patients with missing MMD data at week 12 imputed as non-responders. The odds ratio (OR) compared between the two groups with associated nominal 95% CIs and nominal two-sided *P*-values were reported. Since this study was not powered for secondary endpoints, *P*-values were reported for descriptive purpose only.

The planned sample size was 550 patients. The key assumptions in calculating the sample size were based on prior results from the erenumab global pivotal CM study [28]. A treatment difference in terms of change from baseline in MMD during Weeks 9–12 (primary endpoint) for erenumab 70 mg versus placebo was assumed at − 2.0 days. The common SD of the primary variable was estimated at 6.8. Given a 1:1 randomisation ratio between erenumab 70 mg and placebo, it was required to enroll a total of 550 patients (including a 10% drop out rate) to achieve approximately 90% power to demonstrate the treatment difference of erenumab 70 mg compared with placebo under a two-sided 0.05 alpha level. An interim analysis was performed when 70% patients finished the DBTP or had withdrawn early. The alpha spent at the 70% interim analysis was 0.014 (two-sided); therefore, the alpha for the primary analysis was 0.046 (two-sided). The overall power was maintained at approximately 90% with the planned sample size.

All statistical analyses were performed using SAS® statistical software (SAS Institute Inc., Cary, NC, USA.) version 9.4 or higher.

## Results

### Patient demographics and baseline disease characteristics

In total, 557 patients (out of 791 patients screened) were enrolled and randomly assigned to receive either erenumab (*n* = 279) or placebo (n = 278) (Fig. 1b). Of these randomised patients, 97.8% (n = 545) completed and 2.2% (*n* = 12) discontinued the DBTP (6 in each treatment group) (Fig. 1b).

The overall mean age at baseline was 41.7 (SD ± 10.9) years, and 81.5% (*n* = 454) patients were women. Patient demographics and baseline disease characteristics were well-balanced between the erenumab and placebo groups, except for a slightly higher proportion of women in the placebo group (85.3%; *n* = 237) than in the erenumab group (77.8%; *n* = 217), as gender was not considered a stratification factor for treatment randomisation (Table 1).

**Table 1:** Patient demographics and baseline disease characteristics (Full analysis set)

	**Erenumab 70 mg** ***N* = 279**	**Placebo** ***N* = 278**	**All patients** ***N* = 557**
Age (years), mean (± SD)	41.4 (10.9)	41.9 (10.9)	41.7 (10.9)
Sex, n (%)			
Men	62 (22.2)	41 (14.7)	103 (18.5)
Women	217 (77.8)	237 (85.3)	454 (81.5)
Race, n (%)			
Asian	279 (100.0)	278 (100.0)	557 (100.0)
Chinese	206 (73.8)	215 (77.3)	421 (75.6)
Indian	19 (6.8)	14 (5.0)	33 (5.9)
Korean	22 (7.9)	18 (6.5)	40 (7.2)
Vietnamese	7 (2.5)	8 (2.9)	15 (2.7)
Multiple^b^	2 (0.7)	3 (1.1)	5 (0.9)
Ethnicity, n (%)			
Hispanic or Latino	2 (0.7)	0	2 (0.4)
Not Hispanic or Latino	277 (99.3)	277 (99.6)	554 (99.5)
Not Reported	0	1 (0.4)	1 (0.2)
BMI (kg/m^2^), mean (± SD)	23.3 (4.0) ^c^	23.1 (4.0)	23.2 (4.0)
Monthly migraine days, mean (± SD)	19.1 (5.3)	19.3 (5.6)	19.2 (5.4)
Monthly headache days, mean (± SD)	21.7 (4.2)	22.1 (4.3)	21.9 (4.3)
Monthly moderate and severe headache days, mean (± SD)	16.8 (6.3)	17.2 (6.4)	17.0 (6.4)
Monthly acute headache medication use, days, mean (± SD)	14.1 (8.3)	14.6 (8.2)	14.3 (8.2)
Acute headache medication n (%)			
Yes	262 (93.9)	266 (95.7)	528 (94.8)
None	17 (6.1)	12 (4.3)	29 (5.2)
Age at onset of migraine (years), mean (± SD)	23.3 (10.0)	24.2 (9.6)	23.7 (9.8)
Disease duration of migraine with/without aura (years), mean (± SD)	18.2 (11.9)	17.8 (11.4)	18.0 (11.6)
Aura status, n (%)			
Migraine with aura	197 (70.6)	214 (77.0)	411 (73.8)
Migraine without aura	82 (29.4)	64 (23.0)	146 (26.2)
Prior preventive migraine treatment failure status^a^ n (%)			
Yes	83 (29.7)	83 (29.9)	166 (29.8)
No	196 (70.3)	195 (70.1)	391 (70.2)
Number of prior preventive migraine treatment category n (%)			
None	108 (38.7)	100 (36.0)	208 (37.3)
1	57 (20.4)	62 (22.3)	119 (21.4)
2	41 (14.7)	40 (14.4)	81 (14.5)
3	40 (14.3)	45 (16.2)	85 (15.3)
4	23 (8.2)	18 (6.5)	41 (7.4)
> 4	10 (3.6)	13 (4.7)	23 (4.1)
Number of prior preventive migraine treatment failure n (%)			
None	199 (71.3)	204 (73.4)	403 (72.4)
1	43 (15.4)	46 (16.5)	89 (16.0)
2	31 (11.1)	17 (6.1)	48 (8.6)
3	6 (2.2)	10 (3.6)	16 (2.9)
> 3	0	1 (0.4)	1 (0.2)
Medication overuse^a^ n (%)			
Yes	158 (56.6)	159 (57.2)	317 (56.9)
No	121 (43.4)	119 (42.8)	240 (43.1)

The baseline period for efficacy endpoints is defined as the period between Week − 4 and the day prior to study Day 1

If multiple races have been reported for a patient, the patient was categorised as multiple and in each selected race category. Five patients were identified as multiple races (Asian and White). Asian subgroups were not all-inclusive. The other races were counted only under Asian

*BMI* Body mass index, *SD* Standard deviation

^a^ Reflects the randomisation strata. It might be different from the actual value at baseline

^b^ Patients are both white and Asian

^c^ Data for BMI missing for one patient

At baseline, the mean age at migraine onset was 23.7 (SD ± 9.8), and the mean disease duration of migraine (with or without aura) was 18.0 (SD ± 11.6) years. The mean MMD at baseline was 19.2 (SD ± 5.4), with 19.1 (SD ± 5.3) days in the erenumab group and 19.3 (SD ± 5.6) days in the placebo group. Overall, 37.3% (*n* = 208) patients did not receive any prior preventive treatment. The majority of patients (94.8%, *n* = 528) had used some acute headache medication. The proportion of patients with medication overuse at baseline was 56.9% (*n* = 317). Overall, 29.8% (n = 166) patients had failed prior preventive migraine treatments due to either lack of efficacy or tolerability. The most commonly failed prior preventive medications were calcium channel blockers (flunarizine/verapamil [*n* = 218; 39.1%]), topiramate (*n* = 175; 31.4%), beta-blockers (*n* = 128; 23.0%), and tricyclic antidepressants (*n* = 117; 21.0%).

### Efficacy

#### Monthly migraine days

**Fig. 2:**
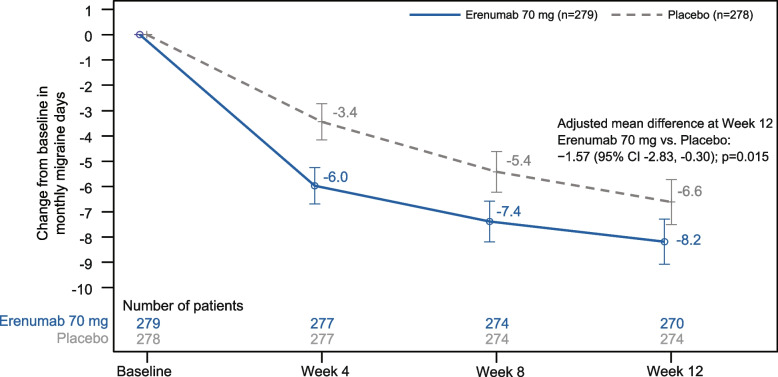
Change from baseline in MMD by treatment and visit (Full analysis set). Adjusted LSM and 95% CIs from the primary analysis model are presented. Note: The primary efficacy endpoint (change from baseline in MMD) was analysed using a generalised linear mixed effects repeated measures model based on observed monthly data during the treatment period. CI, confidence interval; LSM, least-squares means; MMD, monthly migraine days

Treatment with erenumab showed greater adjusted mean reduction from baseline in MMD at each assessment point (Weeks 4, 8 and 12) compared with placebo (Supplementary Table 4). At Week 12, the adjusted mean change from baseline in MMD (primary endpoint) was − 8.2 (SE ± 0.5) days for erenumab and − 6.6 (SE ± 0.5) days for placebo, with a statistically significant difference for erenumab versus placebo (adjusted mean difference vs placebo: − 1.57 [95% CI: − 2.83, − 0.30]; *P* = 0.015) (Fig. 2).

#### Achieving ≥ 50% reduction in MMD

**Fig. 3:**
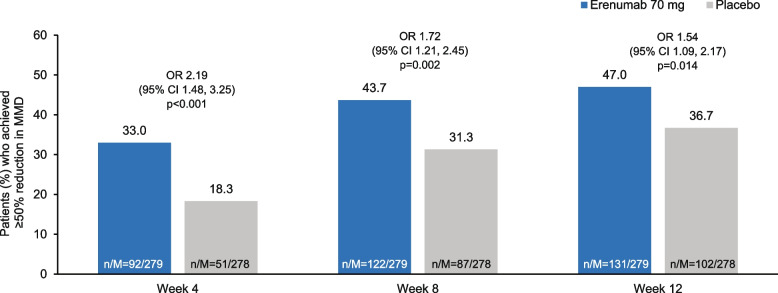
Proportion of patients achieving ≥ 50% reduction from baseline in MMD (Full Analysis Set). *P*-values reported for the dichotomous secondary endpoint (proportion of patients achieving ≥ 50% reduction in MMD) are nominal and should be interpreted with caution. n, number of patients who achieved ≥ 50% reduction in MMD; M, total number of patients in the treatment group with response variable defined. Note: Statistical analysis for the proportion of patients with ≥ 50% reduction from baseline in MMD was done using CMH test adjusting for stratification factor after missing data was imputed (NRI). CI, confidence interval; CMH Cochran–Mantel–Haenszel; MMD monthly migraine day; NRI non-responder imputation; OR, odds ratio

At Week 12, a significantly higher proportion of patients in the erenumab group achieved ≥ 50% reduction in MMD compared with the placebo group (47.0% vs 36.7%). The OR to achieve ≥ 50% reduction in MMD at Week 12 was 1.54 (95% CI: 1.09, 2.17; *P* = 0.014) in the erenumab group versus the placebo group (Fig. 3).

#### Monthly acute headache medication days

The adjusted mean reductions from baseline at Week 12 in monthly acute headache medication days were − 5.34 (SE ± 0.39) in the erenumab group compared with − 4.66 (SE ± 0.39) in the placebo group (adjusted mean difference vs placebo: − 0.67 days [95% CI − 1.76, 0.41]; *P* = 0.223) (Table 2).

**Table 2:** Change from baseline to Week 12 in monthly acute headache medication days and migraine related disability and productivity as measured by the modified migraine disability assessment (mMIDAS)

	**n**		**Adjusted mean change (SE)**		**Comparison of adjusted means** **Test vs Ref**			
	**Erenumab**	**Placebo**	**Erenumab**	**Placebo**	**Difference** **(Test-Ref.)**	**SE**	**95% CI**	**Two-sided** *P***-value**
Monthly acute headache medication days	270	274	− 5.34 (0.39)	− 4.66 (0.39)	− 0.67	0.55	(− 1.76, 0.41)	0.223
mMIDAS score	263	268	− 14.67 (1.20)	− 12.93 (1.19)	− 1.74	1.69	(− 5.06, 1.58)	0.305

The *P*-values reported for the secondary endpoints (monthly acute headache medication days and mMIDAS) are nominal and should be interpreted with caution

The secondary efficacy endpoints (change from baseline in monthly acute headache medication days and mMIDAS) were analysed using a generalised linear mixed effects repeated measures model similar to the primary endpoint

CI, confidence interval; mMIDAS, modified migraine disability assessment; SE, standard error

*n* Number of patients with non-missing value at the corresponding time point of interest

#### Modified migraine disability assessment

The adjusted mean reductions from baseline at Week 12 in the mMIDAS scores were − 14.67 (SE ± 1.20) in the erenumab group compared with − 12.93 (SE ± 1.19) in the placebo group (adjusted mean difference vs placebo: − 1.74 [95% CI − 5.06, 1.58]; *P* = 0.305) (Table 2).

### Safety

98.0% patients (n = 546) received three doses of the study drug, either erenumab 70 mg or placebo. The mean duration of exposure was 11.9 (SD ± 1.2) weeks for the 279 patients who received erenumab. Overall, the proportion of patients with treatment-emergent AEs was comparable between the erenumab (45.5%; *n* = 127) and placebo groups (47.5%; *n* = 132) (Table 3). The most frequently reported treatment-emergent AEs were constipation (erenumab vs placebo: 8.6% [*n* = 24] vs 3.2% [*n* = 9]) and upper respiratory tract infections including nasopharyngitis (9.0% [*n* = 25] in each, erenumab and placebo treatment groups).

**Table 3:** Summary of treatment-emergent AE by preferred term (≥ 1.5% in erenumab group) during the DBTP (Safety analysis set)

	**Erenumab 70 mg** ***N* = 279** **n (%)**	**Placebo** ***N* = 278** **n (%)**
Any AE	127 (45.5)	132 (47.5)
Any treatment-related AE	36 (12.9)	37 (13.3)
AEs leading to study treatment discontinuation	2 (0.7)	2 (0.7)
Any SAE	7 (2.5)	7 (2.5)
Any treatment-related SAE	1 (0.4)	0
SAEs leading to study treatment discontinuation	1 (0.4)	0
Deaths	0	0
*Treatment-emergent AEs (at least 1.5% in erenumab 70 mg group) by preferred term*		
Constipation	24 (8.6)	9 (3.2)
Upper respiratory tract infection	15 (5.4)	20 (7.2)
Nasopharyngitis	10 (3.6)	5 (1.8)
Dizziness	5 (1.8)	12 (4.3)
Pain in extremity	5 (1.8)	1 (0.4)

Preferred terms are sorted in descending order of frequency in erenumab 70 mg column and then alphabetically

A patient with multiple occurrences of an AE under one treatment is counted only once in this AE category for that treatment

A patient with multiple AEs is counted only once in the "at least one AE" row

N, number of patients in the analysis set; n, number of patients reporting at least one occurrence of an adverse event in that class

MedDRA Version 24.0 has been used for the reporting of AEs

*AE* Adverse event, *DBTP* Double-blind treatment phase, *SAE* Serious adverse event, *MedDRA* Medical Dictionary for Regulatory Activities

During the DBTP there were four patients (2 in each group) who discontinued the study drug due to AEs. The AEs leading to discontinuation of the study drug were anaphylactic reaction in two patients receiving erenumab. In this study serious AEs were reported in 2.5% (*n* = 7) patients each in the erenumab and placebo groups (Supplementary Table 5). No deaths were reported during the study.

No clinically significant abnormalities were observed in haematology, biochemistry, ECG, or vital signs in either erenumab 70 mg or placebo groups. The only apparent difference was the proportion of patients with sitting diastolic blood pressure (DBP) < 50 mmHg at any post-baseline visit or decrease from baseline by ≥ 15 mmHg, which was higher in the erenumab-treated group (9.4%; *n* = 26/277) than that in the placebo-treated group (2.9%; *n* = 8/276). None of those changes were considered clinically significant by investigators and no AEs were reported. In the study, the occurrence of clinically notable changes in the sitting heart rate (HR) and systolic blood pressure (SBP) was comparable between the two treatment groups.

At baseline, no patient in the erenumab treatment group had pre-existing anti-erenumab antibodies. Among patients with an on-study result, 1.9% (*n* = 9) developed antibodies against erenumab during the study. The formation of neutralizing antibodies against erenumab during the study was confirmed in 0.4% (*n* = 1) patients treated with erenumab. Overall, 1.7% (*n* = 9) patients developed antibodies against erenumab at any time during the study after the first dose. Of these, neutralizing antibodies against erenumab were developed in 0.2% (*n* = 1) patients.

## Discussion

DRAGON is the first study of erenumab conducted in patients with CM from Asia. This phase 3 study met the primary endpoint of change from baseline in MMD during the last 4 weeks of the 12-week treatment period. Treatment with erenumab 70 mg demonstrated greater reductions in MMD compared with placebo at all assessment time points, from the first post-baseline assessment at Week 4 and sustained throughout the study. Furthermore, analysis of all secondary endpoints confirmed a favourable clinical effect of erenumab over placebo, especially the endpoint of achieving at least 50% reduction in MMD from baseline. Consistent with the results of the phase 3 erenumab study in patients with EM from Asia, the Middle East, and Latin America (EMPOwER study [30]), the DRAGON study demonstrated the efficacy of erenumab in Asian patients with CM and showed that erenumab had a similar safety and tolerability profile. Moreover, the favorable safety profile of erenumab 70 mg in this study was reflected by a high retention rate.

In patients with CM, studies have shown that reduction in headache frequency by 1 day per month is clinically meaningful and significantly improves health-related quality of life [35, 36]. In this phase 3 study, erenumab 70 mg demonstrated a significantly greater reduction in MMD from baseline at Week 12 by 8.2 days compared with 6.6 days with placebo (treatment difference vs placebo: − 1.6 days). Findings from the global pivotal CM study [28] showed that either dose of erenumab, 70 mg or 140 mg, reduced MMD from baseline at week 12 by 6.6 days compared with 4.2 days with placebo (treatment difference vs placebo: − 2.5). The overall reduction in MMD observed in both treatment arms in the DRAGON study was greater than in the global pivotal CM study, which may be related to ethnic or clinical practice differences (e.g. limited use of migraine-specific rescue medications and frequent use of generic painkillers), but the overall treatment effect versus placebo was consistent with the findings from the global pivotal CM study [28].

The baseline demographics and clinical disease characteristics of patients in the DRAGON study were similar to those of patients in the global pivotal CM study [28], with the exception of higher rate of medication overuse (56.9% vs 41.1%, respectively) and the lower rate of prior migraine preventive treatment failures (29.8% vs 67.9%, respectively). The differences between two populations probably reflect the global differences in the clinical practice of CM treatment and availability of migraine-specific preventive medications in Asian countries. However, mean baseline MMD was comparable between the DRAGON study (average of 19 migraine days/month) and the global pivotal CM study (average of 18 migraine days/month) [28], as the CM definition was the same for both studies.

Clinically, ≥ 50% reduction in the mean migraine days per month in response to preventive therapies for migraine is considered a meaningful measure of response and is commonly accepted (i.e., 50% response rate) [35]. Patients with CM treated with erenumab 70 mg in DRAGON study had over 50% higher chance to achieve at least 50% reduction in MMD from baseline as compared to those receiving placebo (OR 1.54; 95% CI: 1.09, 2.17); however, overall proportions of 50% responders were greater in this study than in the global pivotal CM study [28], which was related to the overall greater mean reductions in MMD mentioned above. In the global pivotal CM study [28], the proportions of patients with ≥ 50% reduction in MMD from baseline were smaller, i.e. 40.0% patients in the erenumab 70 mg and 23.0% in the placebo groups, but with greater treatment effect (OR 2.2; 95% CI: 1.5, 3.3). The positive clinical effect was reflected with greater reduction in monthly acute headache medication days and migraine disability as assessed by mMIDAS.

Overall, greater MMD reductions and consequently greater response rates in the DRAGON study than in the global pivotal CM study [28] may reflect greater so-called “placebo effect” in the Asian population. Migraine-specific medication for acute treatment is not widely available in Asian countries, thus patients are more often treated with general painkillers. In the DRAGON study, the higher placebo effect observed did not substantially encumber treatment benefit in patients with CM treated with erenumab 70 mg. Placebo response may contribute to a lack of positive outcomes in clinical studies, especially in those involving pain. In migraine prevention trials, a high variability in placebo effect is observed [37], and there is a need for data on the ethnocultural differences in the placebo effect in migraine prevention. However, the timing of drug trials, not ethnic differences, may play a role, as most of the multinational trials are performed in the Western countries first, then followed by Asian trials. Consequently, strong efficacy evidence based on a large number of prior successful trials may increase the expectations of both patients and physicians. The study was designed with sufficient power to account for the high proportion of treatment-naïve and medication-overuse patients, the high placebo effect in the Chinese/Asian population, and the potentially larger variance in this multiple-national study. Patients were stratified by prior preventive migraine treatment failure and medication overuse status as these were considered to reflect differences in clinical practice between Asian countries, where most patients are treatment naïve and overuse non-migraine specific/acute headache medications. Furthermore, the blinded sample size re-estimation was built in to assess the potential larger variance than expected.

During the study, the majority of (98.0%; *n* = 546) patients received 3 doses of the study drug as planned, suggesting high treatment compliance. The overall incidence of AEs in the DRAGON study was consistent with that in the global pivotal CM study [28]. The safety and tolerability of erenumab were similar to placebo, except for the higher incidence of constipation (8.6% vs 3.2%, respectively), which is a known adverse drug reaction for erenumab. A higher observed incidence of constipation in the DRAGON study than in the pivotal trials was probably related to more awareness of this potential adverse effect for both investigators and patients. With regards to safety of erenumab, there are warnings about constipation with serious complications [31, 32], hypertension [31], and hypersensitivity reactions [31, 32] in the US Prescribing Information and the EU Summary of Product Characteristics [31, 32]. In this study, there were no new safety signals or increased risks with known adverse drug reactions observed with erenumab. The safety profile and tolerability of erenumab were in line with those reported in the EMPOwER study performed mainly in Asian EM patients [30] and the global pivotal CM study [28].

Overall, SAEs (2.5% patients in either treatment groups) were reported as single occurrences (Supplementary Table 5), with one patient in the erenumab group experiencing an anaphylactic reaction that was considered to be related to the study treatment. No deaths were reported during the study. The immunogenicity of erenumab 70 mg observed in the DRAGON study was low and in line with that observed in the global pivotal CM study [28]. Higher proportion of patients with sitting DBP decrease from baseline in the erenumab-treated group than in the placebo group was the only imbalance observed in the vital signs assessments. There is no clear explanation for this phenomenon, as no other changes in SBP or HR were observed; however, it may be speculated that the higher DBP at baseline could be partially related to the “white coat” effect, with further patients’ adaptation to the reality of clinical trial procedures and gradual DBP normalization [38]. The occurrence of clinically notable changes in the sitting HR and SBP was comparable between the two treatment groups.

There are several limitations to this study. First, aura status at baseline was defined as migraine with aura (patient had ever experienced a migraine with aura) and migraine without aura (patient had never experienced any migraine with aura); however, the baseline aura status was self-reported (using the eDiary) rather than being diagnosed by a physician. Many patients may confuse the prodromal symptoms with the aura symptoms within the checklist accompanying the eDiary. It is, therefore, possible that the self-reported migraine prevalence with aura does not reflect the prevalence of diagnosed migraine with aura. The prevalence of migraine with aura in the general population is 12.5% in Taiwan [39] and 29.4% in Korea [40]; however, in this study over 70% patients (Table 1) had reported migraine with aura at baseline. Second, the definition of a migraine day (Supplementary Table 1) in this study was different from the widely accepted definition that includes migraine-specific medication only. If any acute medication was taken during aura, or to treat a moderate or severe headache on a calendar day, the day was counted as a migraine day. This modified definition reflected differences in the clinical practice in Asia, where patients with acute migraine attacks are often treated with painkillers because migraine-specific drugs (e.g., triptans) are not widely available. As a result, this could possibly ‘inflate’ the placebo effect due to the use of rescue medication for every headache (not only migraine), leading to a low treatment effect. Finally, in the DRAGON study only erenumab 70 mg was tested, unlike other migraine studies evaluating both doses of erenumab (70 mg and 140 mg). Erenumab 70 mg was selected as an appropriate efficacious dose in this study based on the global pivotal CM study [28] and the phase 2 EM study in the Japanese population (ClinicalTrials.gov identifier number, NCT02630459) [29]. The differences in efficacy between the two erenumab doses were not substantial [28, 30], with erenumab 70 mg showing a better safety profile. Thus, in the DRAGON study, one dose of erenumab (70 mg) was selected instead of both erenumab 70 mg and 140 mg as in the EMPOwER phase 3 study [30].

In conclusion, the DRAGON study demonstrated that once-monthly subcutaneous erenumab 70 mg is a viable preventive therapy in patients with CM from Asia and is associated with a statistically significant reduction in MMD as well as significant and clinically relevant higher chance of achievement of ≥ 50% reduction in MMD over a period of 3 months compared with placebo, with a favourable safety profile. No new safety signals or increased risks with known adverse drug reactions with erenumab were observed.

## Supplementary Information

**Additional file 1:** **Supplementary Table 1.** Definitions used in the study. **Supplementary Table 2.** Details of eligibility criteria. **Supplementary Table 3.** Patient reported outcome tool: mMIDAS. **Supplementary Table 4. **Change from baseline in MMD by visit, observed,mixed model repeated measures (full analysis set). **Supplementary Table 5. **Patientincidence rates of treatment-emergent SAEs in the study by primary SOC (Safety analysis set).

## Data Availability

The data for the analyses described in this report are available by request from the authors or Novartis Pharma AG, sponsor of this clinical research.

## Abbreviations

AEs: Adverse events
CGRP: Calcitonin gene-related peptide
CHIRA: China Health Insurance Research Association
CI: Confidence interval
CM: Chronic migraine
CTCAE: Common Terminology Criteria for Adverse Events
DBTP: Double-blind treatment phase
EM: Episodic migraine
EU: European Union
GCP: Good Clinical Practice
ICHD-3: International Classification of Headache Disorders, 3rd edition
IRT: Interactive response technology
LSM: Least-squares mean
MedDRA: Medical Dictionary for Regulatory Activities
MMD: Monthly migraine days
mMIDAS: Modified migraine disability assessment
NRI: Non-responder imputation
OLTP: Open-label treatment phase
OR: Odds ratio
SAE: Serious AE
SD: Standard deviation
SE: Standard error
