# Supplementary material for: A phase 3, randomised, placebo-controlled study of erenumab for the prevention of chronic migraine in patients from Asia: the DRAGON study
Source: J Headache Pain. 2022 Nov 21;23(1):146. doi: 10.1186/s10194-022-01514-9 (PMC9676838; doi:10.1186/s10194-022-01514-9)
Supplement: Supplementary file 1 — Additional file 1: Supplementary Table 1. Definitions used in the study. Supplementary Table 2. Details of eligibility criteria. Supplementary Table 3. Patient reported outcome tool: mMIDAS. Supplementary Table 4. Change from baseline in MMD by visit, observed,mixed model repeated measures (full analysis set). Supplementary Table 5. Patientincidence rates of treatment-emergent SAEs in the study by primary SOC (Safety analysis set). [file 10194_2022_1514_MOESM1_ESM.docx]

# Supplementary Tables

**Supplementary Table 1.** Definitions used in the study

| **Term** | **Definition** |
| --- | --- |
| Chronic migraine (CM) | CM is the occurrence of ≥15 headache days per month for more than 3 months, of which ≥8 days per month meet criteria as migraine days (with or without aura). |
| Prior migraine preventive treatments failure | Patients who had prior use of preventive medication but had efficacy or tolerability failures. The number of treatment failures for any patient was based on medication categories. |
| Efficacy failure | Patients who had no meaningful reduction in headache frequency after administration of the respective medication for at least 6 weeks at generally accepted therapeutic dose(s) based on the investigator’s assessment within the last 5 years prior to screening. |
| Tolerability failure | Patients who had documented discontinuation due to adverse events (AEs) of the respective medication at any previous time. |
| Women of childbearing potential | All women physiologically capable of becoming pregnant, unless they are using basic methods of contraception during dosing of investigational drug. |
| Migraine day | A migraine day was defined as any calendar day in which the patient experienced a qualified migraine headache (onset, continuation, or recurrence of the migraine headache). If the patient took any acute medication (simple analgesics [NSAIDs, acetaminophen], combination analgesics, triptans, or ergot derivatives) during aura, or to treat a moderate or severe headache on a calendar day, then it was counted as a migraine day regardless of the duration and pain features/associated symptoms. |
| Headache Day | Any calendar day in which the patient experiences a qualified migraine or a non-migraine headache (initial onset, continuation, or recurrence of the headache lasting ≥4 continuous hours, or a headache of any duration for which acute medication was administered for treatment of headache pain). A headache event that is lasting less than 24 consecutive hours in duration, regardless if it extends into the prior or next calendar day for less than 4 hours even if acute medication is taken on the prior or next calendar day, should be counted as a single headache day. |
| Adverse event | Any untoward medical occurrence (eg, any unfavourable and unintended sign [including abnormal laboratory findings], symptom or disease) in a patient or clinical investigation patient after providing written informed consent for participation in the study. Therefore, an AE may or may not be temporally or causally associated with the use of a medicinal (investigational) product. |
| Treatment-emergent adverse events | Any AE that develops after initiation of study treatment until the end of the last study visit or any event already present that worsens following exposure to the study treatment until the end of the last study visit. |
| Serious adverse event | Any AE [appearance of (or worsening of any pre-existing)] undesirable sign(s), symptom(s) or medical conditions(s)), which meets any one of the following criteria: fatal or life-threatening. |
| Full analysis set | Full analysis set includes all patients who were randomised in the study. Patients were analysed according to their randomised treatment, regardless of the treatment received. Patient demographic and baseline characteristics as well as efficacy analysis was performed on full analysis set. |
| Safety analysis set | Safety analysis set comprised all randomised patients who received at least one dose of investigational product. Patients were analysed based on actual treatment received. Safety analyses were performed based on safety analyses set. |
| Completers | A patient is defined as a completer for the double-blind treatment if the individual had received the Week 8 dose (erenumab or placebo). A patient is defined as completer for double-blind treatment period (DBTP) if the individual had completed scheduled visit in the DBTP or entered in open-label treatment phase. For patients who continued to open-label treatment phase, the cut-off was end of treatment. |
| Response threshold | Achievement of ≥50% reduction in monthly migraine days from baseline was considered to be of high clinical relevance. |

**Supplementary Table 2.** Details of eligibility criteria

| **Inclusion criteria**  Patients eligible for inclusion in this study must meet all of the following criteria:   - Signed informed consent must be obtained prior to participation in the study. - Adult patients aged ≥ 18 to ≤ 65 years upon entry into screening. - History of at least 5 attacks of migraine without aura and/or migraine with visual, sensory, speech and/or language, retinal or brainstem aura according to the IHS Classification ICHD-3 (Headache Classification Committee of the International Headache Society, 3rd edition 2018) based on medical records and/or patient self-report. - History of ≥15 headache days per month of which ≥8 headache days were assessed by the patient as migraine days per month in each of the 3 months prior to screening. - ≥15 headache days of which ≥8 headache days meet criteria as migraine days during the baseline period based on the eDiary calculation (the detailed definition of migraine day can be found in **Supplementary Table 1**). - Demonstrated at least 80% compliance with the eDiary (e.g. must complete eDiary items on at least 23 out of 28 days during the baseline period) based on the eDiary calculation. - History of migraine (with or without aura) for ≥12 months prior to screening according to the IHS Classification based on medical records and/or patient self-report.   **Exclusion criteria**  Patients meeting any of the following criteria are not eligible for inclusion in this study:   - Older than 50 years of age at migraine onset. - History of cluster headache or hemiplegic migraine headache. - Chronic migraine with continuous pain, in which the subject does not experience any pain free periods (of any duration) during the 1 month prior to screening. - Unable to differentiate migraine from other headaches. - Taken an opioid and/or opioid-containing analgesic for any indication during more than 4 days within 1 month prior to the start of the baseline period or during the baseline period. - Taken a butalbital-containing analgesic for any indication during more than 2 days within 1 month prior to the start of the baseline period or during the baseline period. - Prior migraine preventive treatments failure in more than 3 out of the following medication categories: - Category 1: Divalproex sodium, sodium valproate - Category 2: Topiramate - Category 3: Beta blockers (for example: atenolol, bisoprolol, metoprolol, nadolol, nebivolol, pindolol, propranolol, timolol) - Category 4: Tricyclic antidepressants (for example: amitriptyline, nortriptyline, protriptyline) - Category 5: Flunarizine, verapamil, cinnarizine - Category 6: Serotonin-norepinephrine reuptake inhibitors (for example: venlafaxine, desvenlafaxine, duloxetine, milnacipran) - Category 7: Botulinum toxin - Category 8: Lisinopril, candesartan - Category 9: Pregabalin, gabapentin - Category 10: Zonisamide - Category 11: Memantine - Category 12: Pizotifen   Prior migraine preventive treatments failure is defined as efficacy failure or tolerability failure.   - Efficacy failure is defined as "no meaningful reduction in headache frequency after administration of the respective medication for at least 6 weeks at generally accepted therapeutic dose(s) based on the investigator’s assessment within the last 5 years prior to screening." - Tolerability failure is defined as "documented discontinuation due to adverse events of the respective medication at any previous time." - Use of a prohibited medication for migraine prevention within 5 half-lives, or a device or procedure for migraine prevention (e.g. transcranial magnetic stimulation, greater occipital nerve block, invasive or non-invasive neuromodulation) within 1 month prior to the start of the baseline period or throughout the study. - Note: Use of other non-pharmacological treatments and traditional techniques such as acupuncture, traditional and herbal medicine, etc. is in general allowed if the dose/regimen is stable for at least 1 month prior to the start of the baseline phase and also stable throughout the study. - Prior botulinum toxin A treatment in the head/neck region within 4 months prior to the start of the baseline period or during the baseline period. - Active chronic pain syndromes (such as fibromyalgia and chronic pelvic pain). - History of major psychiatric disorder (such as schizophrenia or other psychotic disorders, bipolar disorder, obsessive-compulsive disorder, post-traumatic stress disorder), or current evidence of depression based on a Beck Depression Inventory (BDI)-II total score >24 at screening. Subjects with anxiety disorder and/or major depressive disorder are permitted in the study if they are considered by the investigator to be stable and are taking no more than 1 medication for each disorder. Subjects must have been on a stable dose within the 3 months prior to the start of the baseline period. - History of seizure disorder or other significant neurological conditions other than migraine. (Note: A single childhood febrile seizure is not exclusionary). - History of malignancy of any organ system (other than localized basal cell carcinoma of the skin or in situ cervical cancer), treated or untreated, within the past 5 years, regardless of whether there is evidence of local recurrence or metastases. - History or evidence of any other unstable or clinically significant medical condition, that in the opinion of the investigator, would pose a risk to subject safety or interfere with the study evaluation, procedures or completion. - Human immunodeficiency virus (HIV) infection by history. - Evidence of pre-existing liver condition as defined as any of the following: - Total bilirubin (TBIL) ≥2.0 × upper limit of normal (ULN) or alanine transaminase (ALT) or aspartate aminotransferase (AST) ≥3.0 x ULN, as assessed by the central laboratory at initial screening. - Known history of acute/active hepatitis B virus (HBV) or hepatitis C virus (HCV) within 3 months prior to screening according to local clinical practice. - Myocardial infarction (MI), stroke, transient ischemic attach (TIA), unstable angina, or coronary artery bypass surgery or other revascularization procedure within 12 months prior to screening. - Subject has any clinically significant vital sign, laboratory, or ECG abnormality during screening that, in the opinion of the investigator, could pose a risk to subject safety or interfere with the study evaluation. - Score “yes” on item 4 or item 5 of the Suicidal Ideation section of the Columbia Suicide Severity Rating Scale (C-SSRS), if this ideation occurred in the past 6 months, or “yes” on any item of the Suicidal Behavior section, except for the “Non-Suicidal Self-Injurious Behavior” (item also included in the Suicidal Behavior section), if this behavior occurred in the past 2 years. - Evidence of drug or alcohol abuse or dependence within 12 months prior to screening, based on medical records, patient self-report, or positive urine drug test performed during screening (with the exception of prescribed medications such as opioids or barbiturates). - Pregnant or nursing (lactating) women. - Women of childbearing potential, defined as all women physiologically capable of becoming pregnant, unless they are using basic methods of contraception during dosing of investigational drug. Basic contraception methods include: - Total abstinence (when this is in line with the preferred and usual lifestyle of the subject. Periodic abstinence (eg, calendar, ovulation, symptothermal, post-ovulation methods) and withdrawal are not acceptable methods of contraception. - Female sterilization (have had surgical bilateral oophorectomy with or without hysterectomy), total hysterectomy or tubal ligation at least 6 weeks before taking investigational drug. In case of oophorectomy alone, only when the reproductive status of the woman has been confirmed by follow up hormone level assessment. - Male sterilization (at least 6 m prior to screening). For women in the study, the vasectomized male partner should be the sole partner for that patient. - Barrier methods of contraception: Condom or Occlusive cap (diaphragm or cervical/vault caps). - Use of oral (estrogen and progesterone), injected or implanted hormonal methods of contraception or other forms of hormonal contraception that have comparable efficacy (failure rate <1%), for example hormone vaginal ring or transdermal hormone contraception or placement of an intrauterine device (IUD) or intrauterine system (IUS).   In case of use of oral contraception women should have been stable on the same pill for a minimum of 3 months before taking investigational drug. Women were considered post-menopausal and not of childbearing potential if they have had 12 months of natural (spontaneous) amenorrhea with an appropriate clinical profile (e.g. age appropriate, history of vasomotor symptoms) or have had surgical bilateral oophorectomy (with or without hysterectomy), total hysterectomy or tubal ligation at least 6 weeks ago. In the case of oophorectomy alone, only when the reproductive status of the woman has been confirmed by follow up hormone level assessment is she considered not of child bearing potential. If local regulations deviate from the contraception methods listed above to prevent pregnancy, local regulations apply and will be described in the informed consent form.   - Use of other investigational drugs within 5 half-lives of enrollment, or until the expected pharmacodynamics (PD) effect has returned to baseline, whichever is longer. - History of hypersensitivity to any of the study treatments or its excipients or to drugs of similar chemical classes. - Any prior exposure to investigational or marketed products targeting the CGRP pathway, including previous erenumab studies. - Unlikely to be able to complete all protocol required study visits or procedures, and/or to comply with all required study procedures to the best of the subject’s and investigator’s knowledge. No additional exclusions may be applied by the investigator, to ensure that the study population will be representative of all eligible patients. |
| --- |

**Supplementary Table 3.** Patient reported outcome tool: mMIDAS

| Modified Migraine Disability Assessment Questionnaire (mMIDAS) | The modified MIDAS is a 5-item self-administered questionnaire that sums the number of productive days lost over the past month in the two settings: the workplace and the home. The MIDAS also assesses disability in family, social, and leisure activities. The MIDAS score is the sum of missed days due to a headache from paid work, housework, and non-work (family, social, leisure) activities; and days at paid work or housework where productivity was reduced by at least half. The analysis is based on total score, subscore of absenteeism (items 1, 3, and 5) and subscore of presenteeism (items 2 and 4). The recall period is the past 1 month. The questionnaire takes approximately 5 minutes to complete. |
| --- | --- |

**Supplementary Table 4.** Change from baseline in MMD by visit, observed, mixed model repeated measures (full analysis set)

|  | | **n _________** | | **Adjusted mean change (SE) ________________________** | | **Comparison of adjusted means: Test vs Ref. _________________________________________** | | | |
| --- | --- | --- | --- | --- | --- | --- | --- | --- | --- |
| **Test vs Ref. (Comparison)** | **Visit** | **Erenumab** | **Placebo** | **Erenumab** | **Placebo** | **Difference (Test-Ref.)** | **SE** | **95%CI** | **Two-sided *P*-value** |
| Erenumab 70 mg (N=279) vs Placebo (N=278) | Week 4 | 277 | 277 | −6.0 (0.4) | −3.4 (0.4) | −2.53 | 0.52 | (−3.54, −1.52) | <0.001 |
|  | Week 8 | 274 | 274 | −7.4 (0.4) | −5.4 (0.4) | −1.96 | 0.58 | (−3.10, −0.82) | 0.001 |
|  | Week 12 | 270 | 274 | −8.2 (0.5) | −6.6 (0.5) | −1.57 | 0.64 | (−2.83, −0.30) | 0.015 |

N: The number of patients included in the analysis set. n: number of patients with non-missing value at the corresponding time point of interest.

**Note**: The primary efficacy endpoint (change from baseline in MMD) was analysed using a generalised linear mixed effects repeated measures model based on observed monthly data during the treatment period. A linear mixed effects model includes treatment group, baseline value, stratification factor (random), scheduled visit, and the interaction of treatment group with scheduled visit. Unstructured covariance matrix assumed

CI, confidence interval; MMD, monthly migraine days; SE, standard error

**Supplementary Table 5.** Patient incidence rates of treatment-emergent SAEs in the study by primary SOC (Safety analysis set)

| **Primary system organ class, n (%)** | **Erenumab 70 mg**  **N=279** | **Placebo**  **N=278** |
| --- | --- | --- |
| **Number of patients with at least one SAE** | **7 (2.5)** | **7 (2.5)** |
| Ear and labyrinth disorders | 0 | 1 (0.4) |
| Gastrointestinal disorders | 1 (0.4) | 0 |
| Immune system disorders | 1 (0.4) | 0 |
| Infections and infestations | 1 (0.4) | 2 (0.7) |
| Injury, poisoning and procedural complications | 0 | 1 (0.4) |
| Nervous system disorders | 1 (0.4) | 2 (0.7) |
| Pregnancy, puerperium and perinatal conditions | 1 (0.4) | 1 (0.4) |
| Reproductive system and breast disorders | 1 (0.4) | 0 |
| Surgical and medical procedures | 0 | 1 (0.4) |
| Vascular disorders | 1 (0.4) | 0 |
| **Note:** SOC were presented in alphabetical order. A patient with multiple SAEs within a primary system organ class is counted only once in the total row. A patient with multiple occurrences of an SAE under one treatment was counted only once in this SAE category for that treatment.  MedDRA Version 24.0 was used for the reporting of AEs.  N=Number of patients in the analysis set.; n=Number of patients reporting at least one occurrence of an adverse event in that class; %=n/N * 100  AE adverse event; MedDRA Medical Dictionary for Regulatory Activities; SAEs, serious adverse events; SOC, system organ classes | | |
